# Modeling the Tumor Microenvironment and Cancer Immunotherapy in Next-Generation Humanized Mice

**DOI:** 10.3390/cancers15112989

**Published:** 2023-05-30

**Authors:** Anna Chen, Ines Neuwirth, Dietmar Herndler-Brandstetter

**Affiliations:** Center for Cancer Research, Medical University of Vienna and Comprehensive Cancer Center, 1090 Vienna, Austria; anna.chen@meduniwien.ac.at (A.C.); ines.neuwirth@meduniwien.ac.at (I.N.)

**Keywords:** humanized mice, immuno-oncology, precision oncology, metastasis, PDX, avatar, immune checkpoint blockade, chimeric antigen receptor (CAR), colorectal cancer

## Abstract

**Simple Summary:**

A bottleneck in oncology is the translation of results from preclinical models to the clinics. The rate of anticancer drugs that are effective in preclinical studies but fail in clinical trials is more than 95%. In order to test new immunotherapies and to identify the most effective combination of anticancer drugs, next-generation mouse models have been developed. These “humanized mouse models” support the growth of patient-derived tumors and the development of a human immune system. This review provides an overview of next-generation humanized mouse models and how they can be used to advance precision cancer medicine and immuno-oncology clinical trial design.

**Abstract:**

Cancer immunotherapy has brought significant clinical benefits to numerous patients with malignant disease. However, only a fraction of patients experiences complete and durable responses to currently available immunotherapies. This highlights the need for more effective immunotherapies, combination treatments and predictive biomarkers. The molecular properties of a tumor, intratumor heterogeneity and the tumor immune microenvironment decisively shape tumor evolution, metastasis and therapy resistance and are therefore key targets for precision cancer medicine. Humanized mice that support the engraftment of patient-derived tumors and recapitulate the human tumor immune microenvironment of patients represent a promising preclinical model to address fundamental questions in precision immuno-oncology and cancer immunotherapy. In this review, we provide an overview of next-generation humanized mouse models suitable for the establishment and study of patient-derived tumors. Furthermore, we discuss the opportunities and challenges of modeling the tumor immune microenvironment and testing a variety of immunotherapeutic approaches using human immune system mouse models.

## 1. Introduction

Humanized mice are powerful models for studying human biology, disease and therapeutic interventions. By definition, humanized mice are animals that have either been xenografted with human cells or genetically modified to express human genes. Humanized mouse models aim to more faithfully recapitulate important features of human biology and disease, and therefore represent an important pillar of translational biomedical research and precision medicine-based approaches. In the past 20 years, humanized mice have been used to study the human immune system and infectious diseases (e.g., human immunodeficiency virus (HIV) [1,2], coronavirus disease 2019 (COVID-19) [3,4], hepatitis B and C viruses [5,6], Epstein-Barr virus (EBV) [7] and dengue virus [8]), human erythropoiesis and sickle cell disease [9], malignant diseases (e.g., leukemia [10,11,12], lymphoma [13,14], multiple myeloma [15,16], myelodysplastic syndrome [17], melanoma [18,19]), as well as therapeutic interventions, including cancer immunotherapy [20] and chimeric antigen receptor (CAR) T cell therapy [21].

Although immunotherapy is becoming a cornerstone of modern oncology, complete and durable responses are only observed in a small fraction of cancer patients. For example, results from clinical trials using anti-PD-1 and anti-CTLA-4 checkpoint inhibitors indicate that clinical benefit is limited to only 5% of metastatic colorectal cancer (mCRC) patients who have heavily mutated tumors and tumor-infiltrating T cells [22]. In contrast, 95% of mCRC patients who have a mismatch-repair-proficient, microsatellite instability-low (pMMR/MSI-L) phenotype did not show any clinical benefit. To make matters worse, the success rate of anticancer drugs, i.e., drugs that are effective in preclinical studies as well as clinical trials, is estimated at only 3.4% compared to 20.9% for drugs in all other therapeutic areas of medicine [23]. Hence, there is not only a need for new immunotherapies and more predictive biomarkers, but also preclinical models that accurately reflect the patient’s tumor heterogeneity and tumor immune microenvironment (TIME) [24]. In view of the recent development of a plethora of new immunotherapeutic drugs, it will become more difficult to recruit sufficient numbers of patients to validate all potentially effective combination immunotherapies in clinical trials. Next-generation patient-derived tumor xenograft (PDX) humanized mouse models could be used for preclinical evaluation of the efficacy of combination (immuno)therapies and treatment regimens, for identifying biomarkers of responsiveness, and to stratify cancer patients for clinical trials.

**Figure 1:**
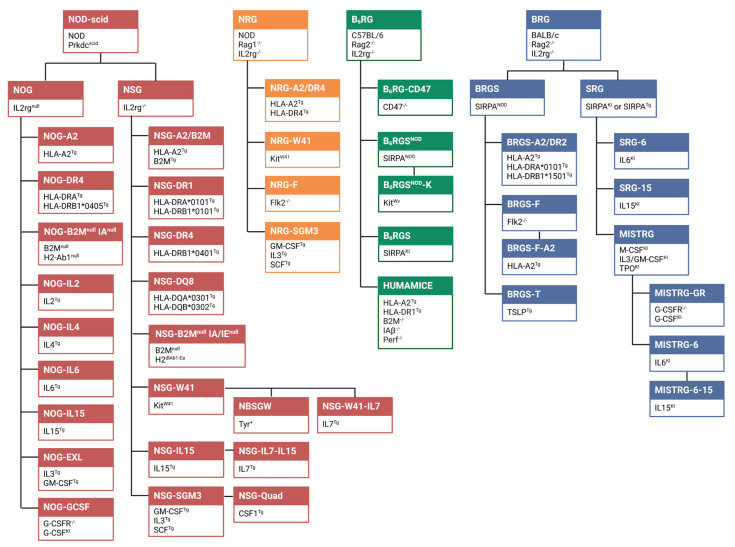
Humanized mouse models for translational cancer research and immunotherapy. The scheme illustrates the genealogical tree of different humanized mouse strains. Details on the characteristics of the individual strains and their usability for generating PDXs and for testing cancer immunotherapies can be found in the text and are summarized in Table 1 and Table 2. Humanized mouse strains that are not commonly used or that are commercial regenerations of established mouse models (e.g., NCG, B-NDG) have not been included in this figure. NRG-A2/DR4 mice are also called “DRAGA” mice. Abbreviations: B_6_RG, C57BL/6 Rag2^-/-^ Il2rg^-/-^; BRG, BALB/c Rag2^-/-^ Il2rg^-/-^; NOD, non-obese diabetic; NOG, NOD SCID Il2rg^null^; NRG, NOD Rag1^-/-^ Il2rg^-/-^; NSG, NOD SCID Il2rg^-/-^; KI, knock-in; SCID, severe combined immunodeficiency; SRG, SIRPA Rag2^-/-^ Il2rg^-/-^; MISTRG, M-CSF IL3/GM-CSF SIRPA TPO Rag2^-/-^ Il2rg^-/-^; Tg, transgene.

Immunocompromised mice are widely used for human tumor xenotransplantation. In 1966, nude mice that lack the majority of mouse T cells were generated [25]. In 1983, BALB/c-^Ighb^ (C.B-17) mice with a spontaneous mutation in the *Prkdc* gene (Prkdc^scid^) were discovered [26]. These mice lacked mouse T and B cells and were called severe combined immunodeficiency (SCID) mice. Backcrossing SCID mice onto a non-obese diabetic (NOD) background (NOD-SCID) resulted in an impaired function of mouse natural killer (NK) cells and a mutation in signal regulatory protein alpha (SIRPA), which conferred high affinity to human CD47 and therefore protected human immune cells from phagocytosis by mouse myeloid cells [27]. However, NOD-SCID mice showed residual NK cell activity and developed lymphomas in old age. To improve PDX engraftment and human immune cell development, NOD-SCID mice with a knockout of the common gamma chain (IL2rg) were generated in 2002 (NSG) [28,29]. Since then, similar highly immunodeficient first-generation humanized mouse strains have been developed, such as NOG [30], BRG [7] and NRG [31] (Figure 1). Similar to the Prkdc^scid^ mutation, knock-out of Rag1 (NRG) or Rag2 (BRG) led to a loss of T and B cells [32]. However, humanized mice on a NOD background (NSG, NOG, NRG) allowed better hematopoietic engraftment compared to other strains, such as C57BL/6 (B_6_RG) or BALB/c (BRG). This was due to a polymorphism in SIRPA, since SIRPA^NOD^ demonstrated enhanced binding to the human CD47 ligand [33]. The expression of human SIRPA (SRG) or SIRPA^NOD^ (BRGS) (Figure 1) was required to prevent phagocytosis of human cells by mouse macrophages and thereby improved human immune cell reconstitution and long-term survival of human immune cell lineages. Providing this “do not eat me signal” in SRG and BRGS mice resulted in human immune system engraftment comparable to that in NSG and NRG mice [14,34,35].

The highly immunodeficient mouse strains NSG, NOG, NRG, BRGS and SRG support the growth of PDXs as well as the development of a human immune system following engraftment with human CD34^+^ hematopoietic stem and progenitor cells (HSPCs). To further improve HSPC and/or PDX engraftment in these mice, numerous genetic modifications have been added to first-generation humanized mouse strains. A genealogical tree of these novel humanized mouse strains is shown in Figure 1. These next-generation humanized mouse models may (1) be more immunodeficient, as they not only lack mouse T, B and NK cells but also have defects in mouse myeloid cell development (e.g., NRG-F, BRGS-F, MISTRG and NOG-GCSF) and/or (2) express human genes in order to promote the growth of PDXs or to improve the development of a diverse and functional human immune system (e.g., MISTRG, NSG-SGM3, NRG-SGM3, NOG-EXL and BRGS-T) [36]. Next-generation humanized mice have been generated to further improve the development and function of human immune cell lineages, thereby enabling preclinical testing of different immunotherapeutic agents. Technological advances, such as CRISPR/Cas9-mediated genome engineering, have facilitated the development of novel next-generation humanized mouse strains, including targeted genomic humanization of mice, so called “knock-in” humanized mice [37,38]. To support ***human T cell development***, humanized mice have been developed that express (**1**) ***human cytokines***, such as interleukin (IL)-7 (NSG-W41-IL7 [39], NSG-IL7-IL15 [40]), IL-15 (SRG-15 [14], NSG-IL15 [41], NSG-IL7-IL15 [40], NOG-IL15 [42]) or thymic stromal lymphopoietin (TSLP; BRGST mice) [43]) or (**2**) ***HLA class I and II molecules***, such as HLA-A2 (NSG-A2 [44], NOG-A2 [45], NRG-A2/DR4 [46], BRGS-F-A2 [47], BRGS-A2/DR2 [48]), HLA-DR1 (NSG-A2/DR1) [49], HLA-DR2 (BRGS-A2/DR2) [48], HLA-DR4 (NRG-A2/DR4 [46], NOG-DR4 [50]) and HLA-DQ8 (NSG-DQ8) [51] (Figure 1).

This review summarizes next-generation humanized mouse models used to establish PDXs of various cancer types and discusses their utility for preclinical testing of different immunotherapeutic strategies and combination treatments to guide clinical trial design.

## 2. Establishing and Modeling Patient-Derived Tumors in Humanized Mice

Cell line-derived xenograft (CDX) models using tumor cells immortalized in vitro from patient tissues have been widely used for preclinical drug development. Tumor cell lines provide an infinite source of biological material, the underlying genetic abnormalities are usually well characterized, and they can be easily cultured and genetically manipulated. However, CDX models often do not accurately mimic tumor heterogeneity and are poorly predictive of efficacy in phase III clinical trials, as evidenced by the high rates of drug attrition in cancer [23,52,53]. Because of the low success rate in establishing cancer cell lines, i.e., only ~10% for colorectal cancer (CRC) [54] or pancreatic cancer [55], the field of oncology has relied on a very limited number of human CDXs per cancer type for preclinical testing of anticancer drugs.

**Figure 2:**
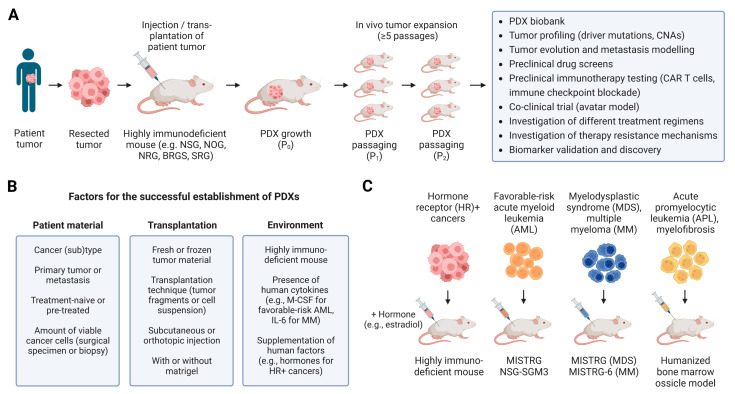
Modeling patient-derived tumors in immunodeficient mice. (**A**) Patient-derived tumor xenograft (PDX) models are established by implanting patient tumor into immunodeficient mice. The PDXs are expanded in vivo by serial passages to establish a PDX biobank. (**B**) Factors that impact the successful establishment of PDXs (initial growth and in vivo propagation for at least 5 passages). (**C**) Immunodeficient mouse models have unique characteristics (e.g., expression of human cytokines) and therefore differently support the engraftment of various cancer types. Abbreviations: AML, acute myeloid leukemia; APL, acute promyelocytic leukemia; CAR, chimeric antigen receptor; CNA, copy number alteration; HR+, hormone receptor-positive; IL-6, interleukin 6; M-CSF, macrophage colony-stimulating factor; MDS, myelodysplastic syndrome; MM, multiple myeloma.

PDXs are generated by transplantation of patient tumors (single cell suspension or tissue fragment) into immunodeficient mice and in vivo propagation for at least 3–5 passages (Figure 2A). PDX models are considered to better recapitulate the histological features, molecular characteristics and intratumor heterogeneity of human cancers, thereby overcoming some of the limitations of CDX models [17,56,57,58,59]. However, ***establishing PDXs*** can take anywhere from several months to more than a year, and depends on a variety of factors, such as the cancer (sub)type, the quantity and quality of the tumor sample, the implantation technique, the implantation site, the immunodeficient mouse strain as well as supplementation of human factors (e.g., hormones for hormone receptor-positive cancers) (Figure 2B) [60,61,62]. In general, metastases engraft better than primary tumors [63] and the more immunodeficient the mouse strain, the greater the likelihood that the patient tumor will engraft and reach stable tumor growth after 5 in vivo passages [64,65,66]. Yet, successful establishment of PDXs has also been achieved when using nude mice that have a lower level of immunodeficiency compared to NSG mice [61,67,68]. Because the mouse host may affect tumor evolution during PDX engraftment and propagation, several studies have extensively analyzed copy number alterations (CNAs) in different PDX models. Guillen and colleagues showed that common driver mutations in breast cancer PDXs were retained in early and late passages with minimal CNAs [69]. By comparing a larger collection of 1127 PDX samples and their originating 324 patient tumors, Woo et al. demonstrated that CNA profiles were conserved during engraftment and passaging of PDXs using CNA inferences based on DNA sequencing [70]. Some CNAs can, however, occur over long-term passaging (e.g., ≥18 passages), and large CNA discordances have even been observed in early passage PDXs, although only in 2.44% of samples [70]. The rare occurrence of large CNA changes suggests that variations observed in PDXs are mostly due to spontaneous selection of tumor clones, rather than a systematic selection pressure by the mouse environment. Another aspect of serial transplantation is the exchange of human stroma by mouse stroma in late passage PDXs, which can affect tumor growth and drug distribution. For instance, increased growth of late versus early passage PDXs has been reported, which may be due in part to the loss of human stroma cells in late passage PDXs [60]. Overcoming these limitations by minimizing passage numbers (a maximum of 5–7 passages has been recommended) [63,71], monitoring CNAs and clonal tumor heterogeneity as well as confirming expected molecular targets is critical to the reproducibility and translatability of preclinical drug screens in PDX mouse models.

Academic and industry institutions (e.g., EurOPDX, PDXNet, PRoXe, NCI-PDMR, PIVOT, COG xenograft repository, LIMORE, MURAL, NIBR PDXE) have established and characterized thousands of ***PDX models generated from a variety of cancer types***, including CRC [61,63,72,73], breast cancer [56,69,74,75], esophagus carcinoma [73], hepatocellular carcinoma [57,62], melanoma [63,76], lung cancer [63,77], prostate cancer [78], gastric cancers [63], and leukemia and lymphoma [79] (Table 1). The success rate of establishing PDXs varies greatly between different tumor types. For example, high success rates of establishing PDXs have been reported for CRC (52–91%), pancreatic cancer (54–100%) and skin melanoma (62–90%) [63,76] whereas lower engraftment rates have been reported for breast cancer (4–86%) and prostate cancer (20.6%) [63,69,78]. In particular, hormone-dependent cancers such as breast and prostate cancers have been shown to have a very low engraftment rate. Accordingly, the huge variability in breast cancer engraftment rate was due to the different types of breast cancer. Engraftment rate for primary estrogen receptor positive (ER+) breast cancers was only 4–20% [63] whereas the engraftment rate for primary triple-negative breast cancer (TNBC) was 30–34% when transplanted subcutaneously, and 60–86% when transplanted orthotopically [63]. In general, orthotopic transplantation of patient tumors yielded higher engraftment rates compared to subcutaneous transplantation [63,80], and primary tumors showed a lower engraftment rate (CRC: 52–75%; TNBC: 30–34%) compared to metastases (CRC: 73–91%; TNBC: 60%) [63]. Of note, the human IL-2 in NOG-IL2 mice may activate tumor-resident T cells in transplanted tumor pieces and reduce the engraftment rate of PDXs in NOG-IL2 compared to NOG mice [81].

Several types of cancer rely on human factors for their growth. For instance, ER+ breast cancers have a very low rate of engraftment and expansion, which is enhanced when estradiol supplementation is provided [74,82,83]. By analyzing 62 TNBCs, Petrosyan et al. found that immunologically “cold” TNBCs engraft at a higher rate than immune cell-enriched “hot” TNBCs [84]. Thus, tumor-infiltrating human immune cells may affect the engraftment rate of some patient-derived tumors, although the mechanisms still need to be experimentally validated. Some ***human hematological neoplasms***, i.e., certain types of leukemia and multiple myeloma, also show low engraftment in immunodeficient mice. For example, the reproducible generation of human acute myeloid leukemia (AML) xenografts is primarily limited to very aggressive “high-risk” cases. Less aggressive, so-called “favorable-risk” AML, which constitute approximately 40% of all AML cases, engraft poorly in immunodeficient mice [85]. Among those favorable-risk AML cases, inv(16) and isolated nucleophosmin mutation (NPM1^mut^) AMLs engrafted with high efficacy in MISTRG but not NSG mice [11] (Figure 2C). Human cytokines, in particular human macrophage colony-stimulating factor (M-CSF), are required for the efficient engraftment of inv(16) AML. NSG-SGM3 mice, also called NSGS mice, express human stem cell factor (SCF), granulocyte-macrophage colony-stimulating factor (GM-CSF) and interleukin 3 (IL-3), and improve the engraftment rate of AMLs from 50% in NSG to 82% in NSG-SGM3 mice [86]. This allowed the identification of different patterns of relapse of AML caused by leukemia stem cells [87]. Improved engraftment of leukemic human hematopoietic cells, acute promyelocytic leukemia (APL) and myelofibrosis samples was achieved by using a humanized bone marrow ossicle xenotransplantation model [88]. Another hematologic malignancy, myelodysplastic syndromes (MDS), has been difficult to engraft in NSG mice, but CD34^+^ HSPCs from MDS patients engrafted well in MISTRG mice [17] (Table 1). Although transient engraftment was observed in NSG-SGM3 mice when HSPCs from MDS patients were co-injected with mesenchymal stromal cells [89], sustained engraftment of MDS in NSG-SGM3 was not observed when using MDS samples from 45 patients [86,90]. This is not surprising, since hematopoietic stem cell maintenance has been shown to be impaired in NSG-SGM3 mice [91,92]. It remains to be seen whether NSG-Quad mice, which are NSG-SGM3 mice that additionally express human M-CSF, will be as suitable a model as MISTRG (Figure 1) [93]. Multiple myeloma (MM) is a hematological neoplasia originating from malignant plasma cells in the bone marrow. Because these malignant plasma cells depend on human IL-6, high engraftment of MM has been achieved in MISTRG-6 humanized mice [16]. Clonal heterogeneity and evolution have major implications for disease progression and relapse in hematologic malignancies. Thus, it will be of great importance to choose humanized mouse models that produce the human factors relevant for the respective PDX entity to avoid animal model-driven changes in tumor clonality and thus biases in preclinical drug development [94,95].

**Table 1:** A selection of immunodeficient mouse models used to generate PDXs.

Mouse Model	Type of PDX	Number of Patients	PDX Engraftment Rate	References
Nude	CRC	85	63.5%	[61]
NOD-SCID, nude	CRC	48	71–74%	[96]
SCID	Uveal melanoma	90	28%	[97]
NOD-SCID	Cervical cancer	22	30.7%	[98]
	CRC (metastatic)	85	87%	[99]
	Esophageal squamous cell carcinoma	25	13.3%	[100]
	Gastric cancer	185	34.1%	[101]
	Leukemia (T-ALL)	19	52%	[102]
	Liposarcoma	56	44.6%	[103]
	NSCLC	75	49%	[104]
NOD-SCID, NSG, NRG	CRC	10	46% (NOD-SCID) 90–91% (NSG, NRG)	[105]
	Ovarian cancer (high-grade serous)	43	76.7%	[106]
NOD-SCID, NSG	PDAC	35	90% (NSG) 40% (NOD-SCID)	[66]
NSG	16 tumor types, such as bladder cancer, breast cancer, CRC, gastric cancer, glioblastoma, HCC, HNSCC, lung cancer, melanoma, ovarian cancer, PDAC, RCC, sarcoma	324 PDXs		[70]
	Breast cancer	83	-	[56]
	CRC	50	54%	[107]
	HNSCC	115	45.2%	[108]
	Nasopharyngeal carcinoma	37	18.9%	[109]
	Testicular cancer	8	38%	[110]
	Melanoma	694	62%	[76]
	Leukemia, lymphoma	138 PDXs	67.5% (B-ALL), 46.7% (T-ALL), 23.2% (AML)	[79]
NSG, NRG	Breast cancer	102	25% (P), 36% (M); 9% (ER^+^ P), 16% (ER^+^ M); 25% (HER2^+^ P), 33% (HER2^+^ M); 29% (TNBC P)	[69]
NSG, NOG	Prostate cancer	48	0%	[111]
NOG	10 tumor types, such as breast cancer, CRC, lung cancer, PDAC and RCC	116	53%	[112]
	Gastric cancer	62	24.2%	[113]
	CLL	7 PDXs	-	[114]
	ALL	60	93.3%	[115]
NOG, NOG-IL2	Metastatic melanoma	21	95% (lower engraftment in NOG-IL2)	[81]
NOG-EXL	AML	26	62%	[116]
NSG, NSG-SGM3	AML	77	82% (NSG-SGM3) 50% (NSG)	[86]
	AML	8	62.5% (NSG-SGM3) 37.5% (NSG)	[117]
NSG, MISTRG	Favorable-risk AML	9	68% (MISTRG) 0–20% (NSG)	[11]
	MDS	31	Higher engraftment in MISTRG	[17]
SRG-6, MISTRG-6	MM	30	Higher engraftment in MISTRG-6	[16]

Abbreviations: ACC, adrenocortical carcinoma; ALL, acute lymphoblastic leukemia; AML, acute myeloid leukemia; CLL, chronic lymphocytic leukemia; CRC, colorectal cancer; ER, estrogen receptor; HCC, hepatocellular carcinoma; HER2, human epidermal growth factor receptor 2; HNSCC, head and neck squamous cell carcinoma; M, metastatic tumor; MDS, myelodysplastic syndromes; MM, multiple myeloma; NSCLC, non-small-cell lung cancer; P, primary tumor; PCa, prostate cancer; PCNA, proliferating cell nuclear antigen; PDAC, pancreatic ductal carcinoma; PD-1, programmed cell death protein 1; PDX, patient-derived xenograft; RCC, renal cell carcinoma; SCLC, small-cell lung cancer; TNBC, triple-negative breast cancer. Abbreviations of humanized mouse strains are listed in the legend of Figure 1.

Numerous studies have demonstrated that PDX models can accurately predict the response to targeted therapies and identify actionable targets in patient subgroups [99,118]. ***Preclinical investigations*** can also be conducted in parallel or sequentially in “avatar” models, when the PDX is established from the tumors of clinical trial participants [63,68,119,120]. Such co-clinical trials showed comparable response rates between PDX models (without a human immune system) and patients, including patients with breast cancer, lung cancer, pancreatic cancer and sarcoma [74,121,122,123,124]. A co-clinical trial of combined MEK and CDK4/6 inhibition in RAS mutant CRC demonstrated therapeutic efficacy, identified biomarkers of response, and revealed mechanisms of resistance in PDXs [125]. In another case, a PDX was generated from a metastatic breast cancer patient who became resistant to treatment with a phosphatidylinositol-3-kinase alpha (PI3Kα) inhibitor [126]. PI3Kα inhibitor therapy led to a loss of PTEN and resistance. By using a PDX nude mouse model, the authors showed that resistance could be avoided by simultaneous blockade of PI3Kα and PI3K p110β. In another case study, eribulin was identified to be effective in an avatar model and was successfully applied to a patient who was initially diagnosed with stage IIA TNBC but experienced metastatic recurrence in the liver [69]. Although the patient’s liver metastases and ascites regressed completely on eribulin, there was isolated progression in bone five months later. In summary, drug screening in avatar models is an innovative but time-consuming and expensive approach that can identify effective treatment options for cancer patients with recurrent disease.

Numerous studies show the benefit of using PDX models, even without a human immune system, to identify determinants of response or resistance to therapies. For example, a collection of 85 PDXs from mCRC patients was established in NOD-SCID mice and used to discover the determinants of resistance following therapy with cetuximab, an anti-epidermal growth factor receptor (EGFR) antibody [99]. The authors identified HER2 as an effective therapeutic target in cetuximab-resistant CRC. Rehman et al. used CRC PDXs and standard-of-care chemotherapy treatment to study CRC cells that become drug-tolerant persisters (DTPs) in NOD-SCID mice [127]. They showed that CRC cells possessed an equipotent capacity to enter a DTP state, and they identified therapeutic opportunities to target DTPs. Additional examples of clinically approved first- and second-generation anticancer drugs that have been successfully tested in preclinical xenograft models has been summarized by Ocana et al. and Byrne et al. [63,128].

In summary, PDX models represent a valuable resource for preclinical drug testing, biomarker validation/discovery, translational research and precision oncology [71,129]. Using PDX models to identify biomarkers of response to therapies has the potential to inform patient enrollment in clinical trials, thereby reducing the cost and time required to study responses in thousands of patients with different (sub)types of cancer.

## 3. Human Immune System Development and Function in Next-Generation Humanized Mice

PDX models represent a useful in vivo platform that accurately predicts the response of therapies that target tumor cells. However, PDX models do not allow study of the interaction of human tumor cells with the human immune system and assessment of the effectiveness of immune cell-targeting cancer therapies. Immunodeficient mice such as NSG, NOG, NRG, B_6_RGS, BRGS and SRG mice not only support the growth of PDXs, but are also capable of developing a diverse human immune system after transplantation of human CD34^+^ HSPCs [7,28,31,34].

Humanized PDX mouse models, which we define here as PDX mice with a human immune system, represent a promising in vivo platform for translational immuno-oncology research, testing cancer immunotherapies, and studying tumor immune escape and immune cell-driven tumor evolution and metastasis [130,131,132]. There is a large variety of humanized mouse strains available and 47 of the most prominent models are shown in Figure 1 using a genealogical tree scheme. Based on the basic immunodeficient models, next-generation humanized mice have been developed that express human cytokines, human leukocyte antigen (HLA) molecules or have other genetic modifications to support human immune cell development and function. Depending on the nature of the preclinical study, the appropriate humanized mouse model should be selected.

The ***development of a diverse and functional human immune system*** in humanized mice depends on three key principles: (1) preventing rejection of human cells (e.g., human tumor cells or human immune cells), (2) enabling of human CD34^+^ HSPC engraftment in the mouse bone marrow niche, and (3) supporting the development and function of human immune cell lineages [133]. An immunodeficient mouse model that lacks mouse T, B and NK cells and that provides phagocytic tolerance (e.g., via SIRPA^NOD^, human SIRPA transgene (SIRPA^Tg^), human SIRPA knock-in (SIRPA^KI^) or mouse CD47^−/−^) is necessary to prevent rejection of transplanted human immune and tumor cells [33]. The corresponding humanized mouse models that fulfill these basic criteria are NSG [28], NOG [30], NRG [31], SRG [14,34], BRGS [134], B_6_RGS^NOD^ [35], B_6_RGS [135] and B_6_RG-CD47 mice [136]. In addition, conditioning of the mouse bone marrow niche is necessary to support long-term engraftment of human CD34^+^ HSPCs. This is accomplished by sublethal irradiation or, less commonly, treatment with myeloablative drugs, such as busulfan [137]. Conditioning of the mouse bone marrow by sublethal irradiation or drug treatment can be avoided by genetic engineering of humanized mice. Introduction of the *Kit^W41^* [138,139] or *Kit^Wv^* mutation (B_6_RGS^NOD^-K [140]) or by expression of human thrombopoietin (THPO) in MISTRG mice using a knock-in (human THPO)/knock-out (mouse THPO) strategy [18] (Figure 1) renders the mouse bone marrow niche susceptible to long-term engraftment of HSPCs without the need for sublethal irradiation. However, a higher proportion of non-irradiated MISTRG humanized mice show poor human immune cell reconstitution (less than 10% of human CD45^+^ cells among all CD45^+^ cells in the blood) compared to irradiated MISTRG mice [18].

**Figure 3:**
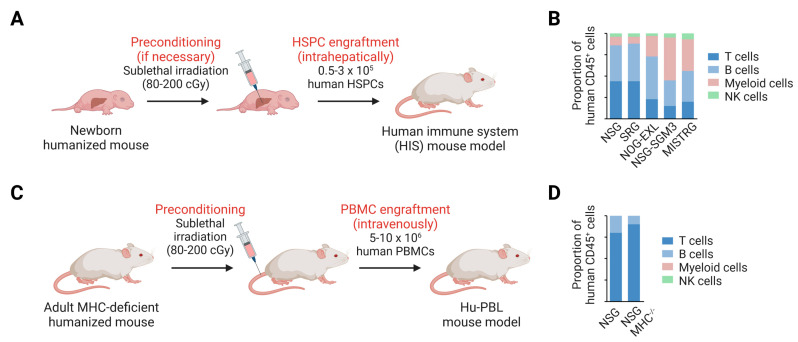
Establishment of human immune system (HIS) mouse models. (**A**) Immunodeficient mice can be reconstituted with a human immune system. For this purpose, newborn mice (1–5 days old) are preconditioned (e.g., sublethal irradiation, busulfan treatment). Some humanized mouse strains, such as NSG-W41, NRG-W41, B_6_RGS^NOD^-K, MISTRG and MISTRG-6 mice, do not need to be preconditioned. Human HSPCs are injected into the liver of newborn mice, which leads to the development of a human immune system. (**B**) The bar graph illustrates the composition of the human immune system in five different humanized mouse models 10–14 weeks post-engraftment with fetal/neonatal HSPCs [14,149,150]. The development, composition and function of the human immune system depends on the humanized mouse model and the source of HSPCs. Fetal and neonatal HSPCs (fetal liver, umbilical cord blood) engraft ≥ 3-fold better than adult HSPCs (bone marrow, G-CSF-mobilized blood) [18,143]. (**C**) MHC-deficient humanized mice can be preconditioned and intravenously engrafted with human PBMCs. (**D**) The bar graph illustrates the composition of human immune system in NSG and MHC-deficient NSG mice 4 weeks post-engraftment with human PBMCs [144]. Abbreviations: cGy, centigray; G-CSF, granulocyte colony-stimulating factor; HSPC, hematopoietic stem and progenitor cell; Hu-PBL, human peripheral blood lymphocyte; MHC, major histocompatibility complex; NK cell, natural killer cell; PBMC, peripheral blood mononuclear cells.

Human CD34^+^ cells can be obtained from umbilical cord, fetal liver, bone marrow or mobilized peripheral blood and different CD34^+^ HSPC engraftment procedures and kinetics have been described [130,141,142]. However, adult CD34^+^ cells have a 3- to 10-fold lower rate of human immune cell reconstitution compared to fetal/neonatal CD34^+^ cells [18,143]. The limited availability of CD34^+^ cells from cancer patients, together with the significantly lower immune cell reconstitution capacity of adult CD34^+^ cells, represents a major obstacle to the generation of PDX mice with an autologous human immune system. Human peripheral blood mononuclear cells (PBMCs) are more readily available from cancer patients than CD34^+^ HSPCs. However, the caveat of engrafting humanized mice with human PBMCs (hu-PBL) is the development of graft-versus-host disease (GVHD), because the transferred mature human T cells have not been educated in the mouse environment. To delay the onset of GVHD, humanized mice that lack mouse major histocompatibility complex MHC class I and/or II molecules (e.g., NSG-B2M^null^ IA/IE^null^, NOG-B2M^null^ IA^null^) [144,145] and/or that express HLAs have been generated (e.g., Rag2^−/−^ Il2rg^−/−^ Prf1^−/−^ B2M^−/−^ HLA-A2/DR1^+^ called “HUMAMICE”) [146]. A delayed onset of GVHD has also been observed in hu-PBL B_6_RG-CD47 mice, which lack mouse CD47 [147]. Another imitation of hu-PBL mouse models is the low number of human B cells and the absence of human myeloid and NK cells one week post PBMC engraftment, due to the lack of de novo generation of human immune cells (Figure 3). Improved human thymopoiesis and T cell responses have been observed in HSPC-engrafted mice that express human HLA class I and II molecules, such as NSG-A2 [148], NOG-DR4 [50], BRGS-A2/DR2 [48] and “HUMAMICE”, with a B_6_RG background [146] (Figure 1).

Cytokines (e.g., IL-6 and IL-7) and B cell-activating factor (BAFF) have been suggested to be important for ***human B cell development and/or survival*** in humanized mice [142]. Indeed, SRG-6 mice that express human IL-6 show not only enhanced thymopoiesis and peripheral T cell engraftment, but also increased class-switched memory B cells and serum immunoglobulin G levels [151]. The effect of human IL-6 on B cells in NSG-IL6 mice remains to be determined [152]. However, replacing mouse BAFF with human BAFF did not improve B cell maturation [153]. In 2014, complete humanization of mouse immunoglobulin loci was achieved, which enables efficient therapeutic antibody discovery [154].

IL-15 is essential for the development, maturation and function of ***NK cells*** and promotes the survival of memory CD8^+^ T cells, including tissue-resident CD8^+^ T cells [155]. Although CD56^bright^ CD16^–^ and CD56^dim^ CD16^+^ NK cell subsets develop in NSG and SRG mice [14], functional NK cells that mediate antibody-dependent cellular cytotoxicity (ADCC), lyse HLA class I negative tumor cells or produce interferon gamma (IFN-γ) upon infection have only been observed in humanized mice that express human IL-2 (NOG-IL2 [156]), human IL-15 (SRG-15 [14], NOG-IL15 [42], NSG-IL15 [41], NSG-IL7-IL15 [40]) or in MISTRG mice, where functional IL-15-expressing human macrophages develop [18].

***Myeloid cells***, such as monocytes, macrophages, dendritic cells and neutrophils, are dependent on several factors for their proper development, survival and function. In particular IL-3, GM-CSF, granulocyte colony-stimulating factor (G-CSF), M-CSF and FMS-like tyrosine kinase 3 (Flt3) ligand have been shown to be important for myeloid cells, and these murine cytokines share less than 80% of their amino acid sequence compared to their human cytokine counterparts [142]. In order to improve the human myeloid compartment, mouse models that express a select combination of these human cytokines have been generated, such as MISTRG [18], NSG-SGM3 [117,149], NRG-SGM3 [157], NOG-EXL [150] and NSG-Quad mice [158]. MISTRG (SRG mice with human M-CSF, IL-3/GM-CSF and THPO) show high engraftment of HSPCs without irradiation, increased development and function of human myeloid cells and NK cells [142], and support engraftment of hematological neoplasms as mentioned in chapter two. NOG-EXL mice (NOG mice with human IL-3 and GM-CSF) show improved development of mature human basophils and mast cells, and enable the study of human allergic responses [150]. NSG-SGM3 mice (NSG mice with human SCF, IL-3 and GM-CSF) show increased human myelopoiesis and functional mast cells, but also an exhaustion of HSPCs [92,149,159]. The BRGF/BRGSF mouse model [160,161] lacks mouse Flt3 and therefore has decreased murine myelopoiesis. Exogeneous supplementation of human Flt3 ligand improved human dendritic cell development. This mouse model could thus be used to test dendritic cell-targeted therapies. In addition, MISTRG-GR [162] and NOG-GCSF mice [163] have been developed, which express human G-CSF and lack mouse G-CSF receptor (G-CSFR), and therefore support human granulocyte development and function.

In summary, a variety of next-generation humanized mouse models have been developed that should enable more authentic reconstruction of the human TIME and preclinical testing of combination (immuno)therapies. A feasible alternative to using human cytokine transgenic or knock-in mouse models is hydrodynamic injection of plasmids encoding human cytokines into immunodeficient mice [133,164].

## 4. Humanized PDX Mouse Models for Preclinical Testing of Cancer Immunotherapies

Currently, there are around 1500 clinical trials assessing different types of cancer immunotherapies, such as immune checkpoint inhibitors (ICIs), antibody-based therapeutics, immunomodulatory drugs and cytokines, CAR cells, therapeutic cancer vaccines and oncolytic viruses (ClinicalTrials.gov; 20 January 2023). Faithful modeling of patient tumor-immune cell interactions and responsiveness/resistance to immunotherapies in humanized PDX mouse models could thus provide critical information on the efficacy of new immunotherapeutic drugs for different cancer types and identify predictive biomarkers. With the development of a large number of new immunotherapeutics, preclinical screening of highly effective combination treatments and prevention of cross-resistance to therapies for specific cancer types will be keys to guide clinical trial design and ensure enrollment of suitable cancer patients. For example, acquired resistance to anti-MAPK therapy has been shown to confer an immune evasive tumor microenvironment and cross-resistance to immunotherapy in melanoma [165,166]. This highlights the need to test for cross-resistance between unrelated therapies and provides a rationale for treating patients with immunotherapy before they acquire resistance to anti-MAPK therapy. Preclinical testing in PDX mouse models has already led to important advancements in cancer therapy and clinical approval of immunotherapeutic agents. For example, anti-CD19 CAR-T cells successfully eradicated CD19^+^ B cell leukemia in PDX NSG mice [167]. The development of next-generation humanized mouse models expressing a variety of human genes that support human immune cell development and function will enable more faithful modeling of human tumor-immune cell interactions and immunotherapies that target different immune cell lineages.

**Table 2:** Humanized PDX mouse models used to test immunotherapeutic approaches.

Mouse Model	Type of PDX	Type of Immunotherapy	Human Immune System Reconstitution	References
NOD-SCID	NSCLC	Non-autologous PBMCs + anti-PD-L1	PBMCs	[104]
NSG	ALL	Anti-CD19 antibody; anti-CD19-TRAIL fusion antibody	No	[168,169]
	Bladder cancer, NSCLC, sarcoma, TNBC	Partially HLA-matched HSPCs + anti-PD-1 antibody	HSPCs	[170]
	CLL	Autologous PBMCs + anti-CD38 antibody	PBMCs	[171]
	Dedifferentiated liposarcoma	Anti-PD-1 antibody	HSPCs	[172]
	Gastric cancer	Mesothelin-specific CAR NK cells	No	[173]
	HCC	Partially HLA-A/B-matched HSPCs + anti-PD-1 or anti-CTLA-4	HSPCs	[174]
	HNSCC	Adoptive transfer of NK cells + anti-PCNA antibody	No	[175]
	Nasopharyngeal carcinoma	Anti-PD-1, anti-CTLA-4	HSPCs	[109]
	Neuroblastoma	Adoptive transfer of activated NK cells + anti-GD2 antibody	No	[176]
	Ovarian cancer	Autologous TILs + anti-PD-1 antibody	TILs	[177]
	TNBC	ACT (cytokine-induced killer cells)	No	[178]
NRG	HNSCC	Radio-immunotherapy: anti-EGFR Ab labeled with ^177^Lu	No	[179]
BRG	Osteosarcoma	GD2- or HER2-targeting BiTE antibody ± anti-PD-1 or anti-PD-L1 antibody	No	[180]
BRGS	ACC, CRC, melanoma, PDAC, SCLC, TNBC	Anti-PD-1 therapy ± TKIs/WNTi/ VEGFi/HDACi	HSPCs	[132]
	CRC	Anti-PD-1 ± cabozantinib, anti-PD-L1 + cobimetinib	HSPCs	[181]
	CRC	Anti-PD-1 antibody	HSPCs	[182]
	ACC	Anti-PD-1 antibody	HSPCs	[183]
NOG-IL2	Metastatic melanoma	ACT	Patient TILs	[184]
NOG, NOG-IL2	Metastatic melanoma	ACT, anti-PD-1 antibody	Patient TILs	[81]
NOG-IL2	Uveal melanoma	Anti-HER2 CAR T cells	No	[185]
NOG-EXL	AML	Co-clinical trial with BETi mivebresib	No	[116]
NOG-EXL, NSG-SGM3	Breast cancer	TLR7/8 agonist activates tumor-infiltrating pDCs	HSPCs	[186]
NSG-SGM3	B-ALL	Anti-PD-1 + anti-CD19 bi-specific T cell engager	HSPCs	[187]
MISTRG	Melanoma (CDX)	Anti-VEGF antibody		[18]
	AML	Anti-CD117 CAR T cells	HSPCs	[188]
	AML	Adoptive transfer of CBFB-MYH11-specific T cells	No	[189]
	HLA-deficient neuroblastoma	Anti-GD2 antibody	HSPCs	[190]
MISTRG-6	DLBCL	Anti-IL-6R antibody	HSPCs	[191]

Abbreviations: Ab, antibody; ACC, adrenocortical carcinoma; ACT, adoptive cell therapy; ALL, acute lymphoblastic leukemia; AML, acute myeloid leukemia; B-ALL, B cell acute lymphoblastic leukemia; BETi, BET inhibitor; BiTE, bispecific T cell engager; CAR, chimeric antigen receptor; CC, cervical cancer; CDX, cell line-derived xenograft; CLL, chronic lymphocytic leukemia; CRC, colorectal cancer; CTLA-4, cytotoxic T lymphocyte-associated protein 4; DC, dendritic cell; DLBCL, diffuse large B cell lymphoma; GC, gastric cancer; GD2, disialoganglioside; GVHD, graft-versus-host disease; HDACi, histone deacetylase inhibitor; HNSCC, head and neck squamous cell carcinoma; HSPC, hematopoietic stem and progenitor cell; Ig, immunoglobulin; IL-6R, interleukin 6 receptor; mAb, monoclonal antibody; MDS, myelodysplastic syndromes; MM, multiple myeloma; NK, natural killer; NPC, nasopharyngeal carcinoma; NSCLC, non-small-cell lung cancer; OC, ovarian cancer; PBMC, peripheral blood mononuclear cell; PCa, prostate cancer; PCNA, proliferating cell nuclear antigen; PDAC, pancreatic ductal carcinoma; PD-1, programmed cell death protein 1; PD-L1, programmed cell death 1 ligand 1; pDC, plasmacytoid dendritic cells; PDX, patient-derived xenograft; RCC, renal cell carcinoma; SCLC, small-cell lung cancer; siRNA, small interfering RNA; TCR, T cell receptor; TIL, tumor-infiltrating lymphocyte; TKIs, tyrosine kinase inhibitors; TNBC, triple-negative breast cancer; VEGF, vascular endothelial growth factor; VEGFi, VEGF inhibitor; WNTi, Wnt signaling pathway inhibitor. Abbreviations of humanized mouse strains are listed in the legend of Figure 1.

In the past decade, tumor-infiltrating myeloid cells, including tumor-associated macrophages (TAMs), have emerged as critical regulators of the TIME [192,193]. They promote metastasis and therapy resistance, and M2 TAMs and polymorphonuclear myeloid-derived suppressor cells (PMN MDSCs) are associated with poor clinical prognosis in many cancer types [194,195]. TAMs can also produce immunosuppressive molecules, such as IL-10, transforming growth factor (TGF)-β, inhibitory checkpoint molecules like programmed-death ligand 1 (PD-L1) as well as angiogenic factors, such as vascular endothelial growth factor (VEGF) [196,197]. Efficient development of human myeloid cell lineages in humanized mice is therefore important to faithfully model an immunosuppressive tumor microenvironment, angiogenesis and metastasis.

**Figure 4:**
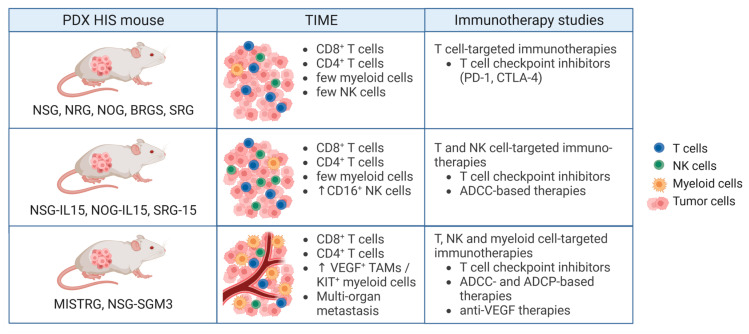
Cancer immunotherapy in humanized PDX mouse models. Efficient and functional development of human immune cell lineages depends on the humanized mouse model. This also influences the composition of the TIME and the suitability of investigating different types of immunotherapies [14,18,83]. Abbreviations: ADCC, antibody-dependent cellular cytotoxicity; ADCP, antibody-dependent cellular phagocytosis; CTLA-4, cytotoxic T lymphocyte-associated antigen 4; HIS, human immune system; KIT (also known as c-kit, CD117 or stem cell factor receptor); NK, natural killer; PD-1, programmed cell death protein 1; TAM, tumor-associated macrophage; TIME, tumor immune microenvironment; VEGF, vascular endothelial growth factor.

Using a ***humanized CDX mouse model***, human macrophages were shown to infiltrate melanoma CDXs in humanized MISTRG mice and promote tumor growth [18] and metastasis [19]. Increased tumor growth was dependent on human VEGF produced by tumor-infiltrating human macrophages. Treatment with the anti-human VEGF antibody bevacizumab or depletion of human phagocytic cells with clodronate reversed the increased tumor growth in MISTRG humanized mice (Table 2, Figure 4). However, the development of functional human macrophages in HSPC-engrafted MISTRG mice also caused anemia and loss of reconstitution over time, which limits the time available to perform experiments and evaluate the efficacy of therapies [18]. Human KIT^+^ myeloid cells were shown to facilitate metastasis in melanoma CDX NSG-SGM3 but not NSG humanized mice, which lack human factors that promote efficient human myeloid cell development [92,198]. In both, MISTRG and NSG-SGM3 humanized mouse models, the melanoma-infiltrating myeloid cells were transcriptionally similar to myeloid cells found in melanoma patients [19,198]. Using a NOD/SCID humanized mouse model, Su et al. demonstrated that CCL18 usually produced by TAMs contributes to epithelial-mesenchymal transition (EMT) and metastasis in a breast cancer CDX model [199]. However, when analyzing tumor-infiltrating plasmacytoid dendritic cells (pDCs), the intratumoral frequency of pDCs was dependent on the tumor type rather than on the humanized mouse model (NOG vs. NOG-EXL) [186]. NOG and NOG-EXL humanized mice equally supported human myeloid cell infiltration into three different human ovarian and breast cancer CDXs.

In ***humanized PDX mouse models***, the frequency of human CD45^+^ cell infiltration into tumors was dependent on the individual PDX, as only 6 of 11 microsatellite stable (MSS) CRC PDXs showed an infiltration with human CD45^+^ cells of greater than 0.1% [132]. TNBC PDXs in NSG humanized mice showed infiltration of human T cells and CD68^+^ myeloid cells and generated lung metastases similar to those of TNBC patients [200]. In a study by Scherer et al., human ER+ breast cancer PDX NSG-SGM3 humanized mice could recapitulate the lymphocyte-excluded and myeloid-rich TIME of ER+ breast cancer patients [83]. Similarly, more CD33^+^ myeloid cells infiltrated a melanoma PDX in humanized NSG-SGM3 compared to NSG mice [201], and a neuroblastoma PDX MISTRG humanized mouse model recapitulated the lack of activated human NK cells observed in neuroblastoma patients [190]. Interestingly, even NSG mice without a human immune system partially recapitulated patient melanoma genotype-associated histopathological features [202] and allowed the analysis of metastasis-associated features of orthotopically engrafted CRC PDXs [203]. Proper development of human myeloid cells will pave the way for testing myeloid-specific phagocytosis checkpoint inhibitors. For example, blockade of the phagocytosis checkpoint SIRPA, which binds to CD47 that is upregulated on tumor cells [204], has been shown to enhance myeloid cell-dependent killing of Burkitt’s lymphoma through antibody-dependent cellular phagocytosis in SRG mice [205]. Together, these studies indicate that humanized PDX mouse models are able to recapitulate features of the patient’s TIME and may therefore be useful for preclinical testing of the efficacy of immune-based therapies and the discovery of predictive biomarkers.

***Immunotherapy in humanized PDX mouse models*** includes anti-PD-1 therapy of bladder cancer [170], hepatocellular carcinoma (HCC) [174], melanoma [206], non-small-cell lung cancer (NSCLC) [170,207,208], autologous renal cell carcinoma (RCC) [209], sarcoma [170,172] and TNBC [200] in humanized NSG mice, as well as adrenocortical carcinoma [183], microsatellite instable (MSI) and MSS CRC [132,182], melanoma [132] and TNBC [132] in humanized BRGS mice (Table 2). Anti-PD-1 therapy in these models reduced tumor growth in approximately 75% of PDXs and led to changes in tumor-infiltrating lymphocytes and IFN-γ-related genes. In the case of TNBC PDXs, therapy responsiveness seemed to correlate with PD-L1 expression on tumor cells [200]. By using TNBC CDX BRGS humanized mice, Tentler et al. showed that RX-5902, a novel β-catenin modulator, improved the responsiveness to PD-1 or CTLA-4 inhibitor therapy [210]. Using NSG mice, HLA-A-matched CD34^+^ cells, fetal thymus and DNA-based vectors to provide human cytokines, Somasundaram et al. demonstrated that co-recruitment of mast cells and forkhead box P3 (FoxP3)^+^ regulatory T cells into melanomas was associated with resistance to anti-PD-1 therapy [211]. Combination of anti-PD-1 therapy with the tyrosine kinase inhibitors sunitinib or imatinib led to the depletion of mast cells and complete regression of tumors. By using MSI CRC patient-derived organoids orthotopically injected into NSG humanized mice, Kücükköse et al. showed that anti-PD-1 or anti-CTLA-4 therapy reduced primary CRC size and eradicated liver metastases but had no effect on peritoneal metastases [212]. This humanized PDX mouse model enabled multiorgan metastasis and suggested that high levels of immunosuppressive cytokines in ascites might promote resistance of peritoneal metastases to ICI therapy.

A major challenge for immuno-oncology in the coming years will be to identify the most ***effective combination immunotherapies*** for each cancer type and to identify treatment regimens that prevent cross-resistance between different therapies. So far, combination therapy of the CD19-directed bi-specific T cell engager blinatumomab and anti-PD-1 antibody pembrolizumab has shown improved survival in B-cell acute lymphoblastic leukemia PDX NSG-SGM3 mice [187]. Combination of anti-PD-1 antibody with histone deacetylase inhibitors has also shown successful treatment outcomes in breast cancers [182]. Cabozantinib, a multi-tyrosine kinase inhibitor, sensitized MSS CRC PDXs to anti-PD-1 therapy by increasing the frequency of cytotoxic and IFN-γ^+^ T cells in BRGS humanized mice [181]. This treatment also decreased the expression of T-cell immunoglobulin and mucin-domain containing-3 (TIM-3), which is an inhibitor immune checkpoint and a marker of exhaustion on CD8^+^ T cells [213,214].

Another critical component of the TME are cancer-associated fibroblasts (CAFs), which can facilitate metastasis, therapeutic resistance and dormancy [215]. CAFs consist of different subpopulations with distinct phenotypes, functions and origins. Because the interaction of CAFs with tumor and immune cells critically shapes the TME and response to (immuno)therapies, it is important to properly model CAFs in humanized PDX mice. However, human CAFs that are present in the primary tumor graft are gradually replaced by mouse fibroblasts during in vivo passaging [105,216]. In a proof-of-principle study, Weinberg and colleagues used NOD-SCID mice to demonstrate that mesenchymal stromal cells (MSCs) promote breast cancer metastasis via the chemokine CCL5 [217]. Human CAFs were also shown to drive tumor progression in NSCLC-PDX NOD-SCID mice [218], and CAF subsets have been associated with (chemo)therapy resistance in TNBC and NSCLC PDX mouse models [104,219]. Of note, it may be challenging to support the long-term survival of transferred human MSCs [90], although co-injection of patient tumors and MSCs into 3D scaffolds improves the maintenance of transferred human MSCs.

Taken together, these studies demonstrate the usefulness of humanized PDX mouse models for preclinical screening of the efficacy of immunotherapies in different cancer types and subtypes.

## 5. Conclusions and Future Perspectives

In the past decade, numerous next-generation humanized mouse models have been generated, each with distinct improvements to enable the development of a more diverse and functional adaptive or innate immune system, or to facilitate the generation of PDXs from a wider variety of cancer types. This will ultimately accelerate preclinical screening of new immunotherapies and the discovery of biomarkers and effective treatment regimens and combination therapies. However, the unique properties (improvements and shortcomings) of each humanized mouse model must be considered in order to faithfully reconstruct the patient’s TIME and to obtain meaningful results that can be transferred to the clinic (Table 2). For example, CAR-T cell, CAR-NK cell or tumor-infiltrating lymphocytes (TILs) therapies should be performed in humanized mouse models expressing human cytokines that support T or NK cell survival and function, such as IL-2 or IL-15 [184]. In addition, adoptive T cell therapy (e.g., CAR-T cells, TILs) is routinely being performed in mice devoid of a diverse, endogenous human immune system, therefore underestimating the impact of the immunosuppressive tumor microenvironment on the function and persistence of the adoptively transferred T cells, or the ability of these T cells to infiltrate PDXs.

Despite advances in the field, some challenges and limitations remain regarding the translational value of next-generation humanized mice, such as HLA class I and II incompatibility between PDX and HSPC, and the expensive and time-consuming generation of humanized PDX mouse models. Nonetheless, the research studies highlighted in this review demonstrate the potential of humanized PDX mouse models to address key questions in precision immuno-oncology and to assist in identifying effective combination (immuno)therapies and treatment regimens for different cancer types and subtypes.

## Data Availability

Not applicable.
